# No difference in ACL revision rates between hamstring and patellar tendon autograft in patients with ACL‐R and a concurrent meniscal injury irrespective of meniscal treatment

**DOI:** 10.1002/ksa.12592

**Published:** 2025-01-23

**Authors:** Johan Högberg, Lina Petersson, Bálint Zsidai, Alexandra Horvath, Riccardo Cristiani, Kristian Samuelsson, Eric Hamrin Senorski

**Affiliations:** ^1^ Sportrehab Sports Medicine Clinic Gothenburg Sweden; ^2^ Sahlgrenska Sports Medicine Center Gothenburg Sweden; ^3^ Unit of Physiotherapy, Department of Health and Rehabilitation Institute of Neuroscience and Physiology, Sahlgrenska Academy University of Gothenburg Gothenburg Sweden; ^4^ Department of Orthopaedics Institute of Clinical Sciences, Sahlgrenska Academy University of Gothenburg Gothenburg Sweden; ^5^ Department of Molecular Medicine and Surgery Section of Sports Medicine, Karolinska Institute Stockholm Sweden; ^6^ Stockholm Sports Trauma Research Center (SSTRC), FIFA Medical Centre of Excellence Stockholm Sweden; ^7^ Department of Orthopaedics Sahlgrenska University Hospital Mölndal

**Keywords:** anterior cruciate ligament, graft choice, KOOS, meniscus, revision

## Abstract

**Purpose:**

The aims of this study were to compare (1) the rate of anterior cruciate ligament (ACL) revision and (2) subjective knee function using the Knee injury and Osteoarthritis Outcome Score (KOOS) between isolated ACL reconstruction (ACL‐R) and ACL‐R and concurrent meniscal injury, based on graft selection and meniscal treatment.

**Methods:**

Data from the Swedish National Knee Ligament Registry were extracted in November 2022 for patients who underwent primary ACL‐R. Patients were divided into two main groups based on graft choice: hamstring tendon (HT) or patellar tendon (PT) autograft, with four meniscal sub‐groups: no injury, resection, repair or left in situ. The primary outcome was the rate of ACL revision within 5 years of primary ACL‐R, and the secondary outcome was subjective knee function measured with the mean KOOS subscale scores and the rate of patients achieving a patient‐acceptable symptom state (PASS) at the 1‐, 2‐ and 5‐year follow‐up.

**Results:**

The analysis of ACL revision was performed on 45,656 patients, and 7639 patients for the analysis of subjective knee function. The overall rate of ACL revision was 2.4% and 4.9% at 2 and 5 years, respectively. There were no differences in the rate of ACL revision within 5 years of primary surgery irrespective of graft choice or meniscal injury treatment. Patients with ACL‐R and concurrent meniscal resection or meniscal injury left in situ achieved a PASS at the 1 (*∆* = −11.3% to −29.5%), 2 (*∆* = −12.7% to −40.3%) and 5‐year (*∆ *= −12.0% to −30.6%) follow‐up to a greater extent when receiving HT autograft compared to PT autograft.

**Conclusion:**

Graft selection was not associated with ACL revision in patients with ACL‐R and concurrent meniscal injury, regardless of meniscal injury treatment. Superior subjective knee function was reported by patients who underwent ACL‐R with HT autograft compared with PT autograft where the injured meniscus was resected or left in situ.

**Level of Evidence:**

Level III.

**Abbreviations**
ACL: anterior cruciate ligament
ACL‐R: anterior cruciate ligament reconstruction
BMI: body mass index
CI: confidence interval
cm: centimeters
HR: hazard ratio
HT: hamstring tendon
kg: kilogram
KOOS: Knee injury and Osteoarthritis Outcome Score
KOOS‐ADL: subscale activities in daily living
KOOS‐P: subscale pain
KOOS‐QoL: subscale quality of life
KOOS‐S: subscale symptoms
KOOS‐SR: subscale sports and recreational
LCL: lateral collateral ligament
MCL: medial collateral ligament
n: number of patients
n.s: non‐significant
PASS: patient acceptable symptom state
PCL: posterior cruciate ligament
PROMs: patient‐reported outcome measurements
PT: patellar tendon
SNKLR: Swedish National Knee Ligament Registry

## INTRODUCTION

An isolated anterior cruciate ligament (ACL) injury is considered rare, with 50%–79% of ACL injuries being reported with a concurrent meniscal injury [15, 19, 48]. Interestingly, the occurrence of meniscal injuries has been reported to be greater in patients with longer time from injury to ACL reconstruction (ACL‐R) [8, 19, 26, 34]. The ACL controls anterior translation and anterolateral rotation of the tibia [12]. In vitro studies have revealed an increased rotatory knee laxity in ACL‐deficient knees with concurrent meniscal injuries compared to cases of isolated ACL injury [25, 29]. A systematic review [18] reported that medial meniscus tear or resection increased the anterior tibial displacement, while the lateral meniscus tear or resection increased the rotation in the ACL‐deficient knee. The persistence of a pivot shift has been observed despite undergoing ACL‐R, potentially attributed to unrepaired meniscal tears [23]. Consequently, the presence of a concurrent meniscal injury should not be overlooked in patients with ACL injury due to the menisci's role in stabilising the knee joint.

Limitations in knee function and persistent knee laxity are frequently reported after ACL‐R [24]. Patients with greater preoperative knee laxity have been reported to present with persistent postoperative pivot shift [46]. Graft choice can have an important impact on postoperative residual laxity as hamstring tendon (HT) autografts have been associated with greater postoperative anterior and rotatory knee laxity compared with patellar tendon (PT) autografts [10, 21]. In addition, the semitendinosus and gracilis tendons, which constitute the HT autograft, play a role in decreasing excessive knee valgus, thereby partly contributing to knee joint stability [22]. Hence, patients with greater knee laxity preoperatively might experience improved postoperative outcomes in terms of knee‐joint laxity by opting for a PT autograft over an HT autograft [42].

Outcomes between the HT and PT autograft are comparable in subjective knee function, but an increased incidence of graft failure has been reported for HT autograft at medium‐term follow‐up of 5 years [50]. Furthermore, the rate of isolated meniscal repair failures has been reported to be greater in young females treated with HT autografts compared to PT autografts after their ACL‐R (20.7% vs. 2.4%) [36], potentially attributed to a greater knee laxity for patients treated with HT autografts compared to PT autografts [38]. As both the choice of autograft [10, 21, 38] and meniscal status [18] influence knee laxity, is it important to further add the meniscal treatment into the algorithm when comparing outcomes between HT and PT autografts.

The aims of this study were to compare (1) the rate of ACL revision and (2) subjective knee function using the Knee injury and Osteoarthritis Outcomes Score (KOOS) in patients with isolated ACL‐R and ACL‐R with concurrent meniscal injury, based on (1) graft selection and (2) meniscal injury treatment.

The hypothesis was that patients with a meniscal injury left in situ, or resected would have a greater rate of ACL revision and worse subjective knee function when treated with HT autograft compared to PT autograft.

## MATERIALS AND METHODS

Ethical approval for this study was granted by the Swedish Ethical Review Authority under registration number 2022‐00913‐01. The study adhered to the guidelines of the Helsinki Declaration [2]. This research was reported according to the Reporting of studies Conducted using Observational Routinely‐collected health Data (RECORD) statement [5]. Data were queried from the Swedish National Knee Ligament Registry (SNKLR), a nationwide population‐based database. Established on 1 January 2005, the SNKLR encompasses over 90% of annual primary ACL‐Rs performed in Sweden, with approximately 47,000 patients registered since inception [14]. The registry employs a web‐based protocol, automatically recording sex and age based on the Swedish social security number. The protocol comprises two sections: one for patients and one for surgeons.

The patient section entails data before ACL‐R and at intervals of 1, 2, 5 and 10 years after ACL‐R. Data encompasses demographic information and patient‐reported outcome measurements (PROMs), including the KOOS [35] and the European Quality of Life‐5 Dimensions (EQ‐5D) [1].

The surgeon section includes details on the date of injury, activity at injury, prior surgeries, intraoperatively identified concurrent injuries, surgical techniques, graft selection and graft diameter. In cases of ACL revision or subsequent surgeries for other reasons, separate data entries are registered and linked to the primary ACL‐R procedure.

The SNKLR adheres to Swedish data security legislation safeguarding the integrity of enrolled patients [3]. Participation in the SNKLR is voluntary, and all patients receive information about the registry and may decline participation without reason.

### Population

Data was extracted from SNKLR in November 2022. Patients registered in the SNKLR who underwent primary ACL‐R were assessed for eligibility. Inclusion criteria were as follows: Patients aged ≥15 years with a minimum 5‐year follow‐up and primary ACL‐R using ipsilateral HT autograft or PT autograft. For the second outcome, that is, to investigate the difference in subjective knee function, only patients with complete data for KOOS at 1, 2 and 5 years after ACL‐R were included. Exclusion criteria were prior contralateral ACL injury/reconstruction, autografts sourced from the contralateral side, treated with both concomitant meniscal resection and repair, and concomitant fracture, vascular or nerve injuries. Additionally, patients with concomitant posterior cruciate ligament injuries and/or surgical interventions for medial or lateral collateral ligament injuries were excluded.

### Definition of groups

**Figure 1:**
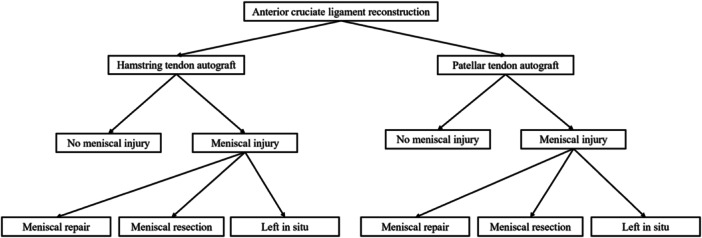
Definition of groups.

Upon inclusion, patients were stratified into eight groups based on (1) graft choice: HT autograft or PT autograft, (2) concurrent meniscal injury (yes/no) and (3) meniscal injury treatment: resection, repair or left in situ, within each respective graft group (Figure 1).

### Outcomes

The primary outcome was the rate of ACL revision within 5 years of primary ACL‐R, and the secondary outcome was subjective knee function measured with the mean KOOS subscale scores and the rate of patients achieving a patient‐acceptable symptom state (PASS) 1, 2 and 5 years after ACL‐R. The follow‐up period for the primary outcome, that is, ACL revision, commenced at the date of the index ACL‐R and continued until either ACL revision occurred or reached 5 years after ACL‐R. Information on ACL revision was obtained from the SNKLR.

### Patient‐reported outcome measures

The KOOS was developed to assess symptoms and subjective knee function subsequent to knee injury [35]. The KOOS consists of five independent subscales with 4–17 items per subscale. Patients respond to a Likert scale with five possible answers ranging from 0 (*no problems*) to 4 (*extreme problems*). Each subscale is transformed to a 0–100 scale with scores evaluated between 0 (*indicative of the poorest outcome*) to 100 (*indicative of the most favourable outcome*). There are five subscales: pain (KOOS‐P), symptoms (KOOS‐S), activities of daily living (KOOS‐ADL), sports and recreation (KOOS‐SR) and quality of life (KOOS‐QoL) [35].

To ascertain the clinical significance of the KOOS, the concept of PASS was applied at 1, 2 and 5 years following ACL‐R. Thresholds of achieving PASS were calculated by Muller et al. [28] by asking the patients the following question: ‘*Taking into account all the activity you have during your daily life, your level of pain, and also your activity limitations and participation restrictions, do you consider the current state of your knee satisfactory?*’ The calculated PASS threshold used were the following: 88.9 for KOOS‐P, 57.1 for KOOS‐S, 100 for KOOS‐ADL 75.0 for KOOS‐SR and 62.5 for KOOS‐QoL [28]. The test–retest reliability of the KOOS has yielded intraclass coefficient (ICC) values ranging from 0.70 to 0.95, indicative of consistent measurement reliability for assessing short and long‐term knee symptoms and function [7].

### Statistical analysis

Statistical analyses were conducted using the Statistical Analysis System Software (SAS Statistics for Windows, version 9.4, SAS Institute). Demographic data were presented using counts (*n*) and proportions (%) for categorical variables, whereas continuous variables were described using means with standard deviation, as well as median with minimum and maximum values. To compare differences in demographics, Fisher's exact test was used for dichotomous variables, and an independent *t* test was used for continuous variables.

Survival analysis was performed using a univariable Cox proportional hazard regression, expressed as hazard ratio (HR) with 95% confidence intervals (CIs) for ACL revision between the groups. Sex, age, body mass index (BMI) and type of meniscal injury (i.e., medial or lateral) were considered as covariates in the multivariable analysis. Independent *t* test was used to compare the groups for differences in KOOS and Fisher's exact test for investigating PASS for each subscale. A *p* value of 0.05 and a CI of 95% were set. Cohen's *d* was applied to assess the effect size of the differences according to the following reference values: 0.20 = small, 0.50 = medium and 0.80 = large [6].

## RESULTS

**Figure 2:**
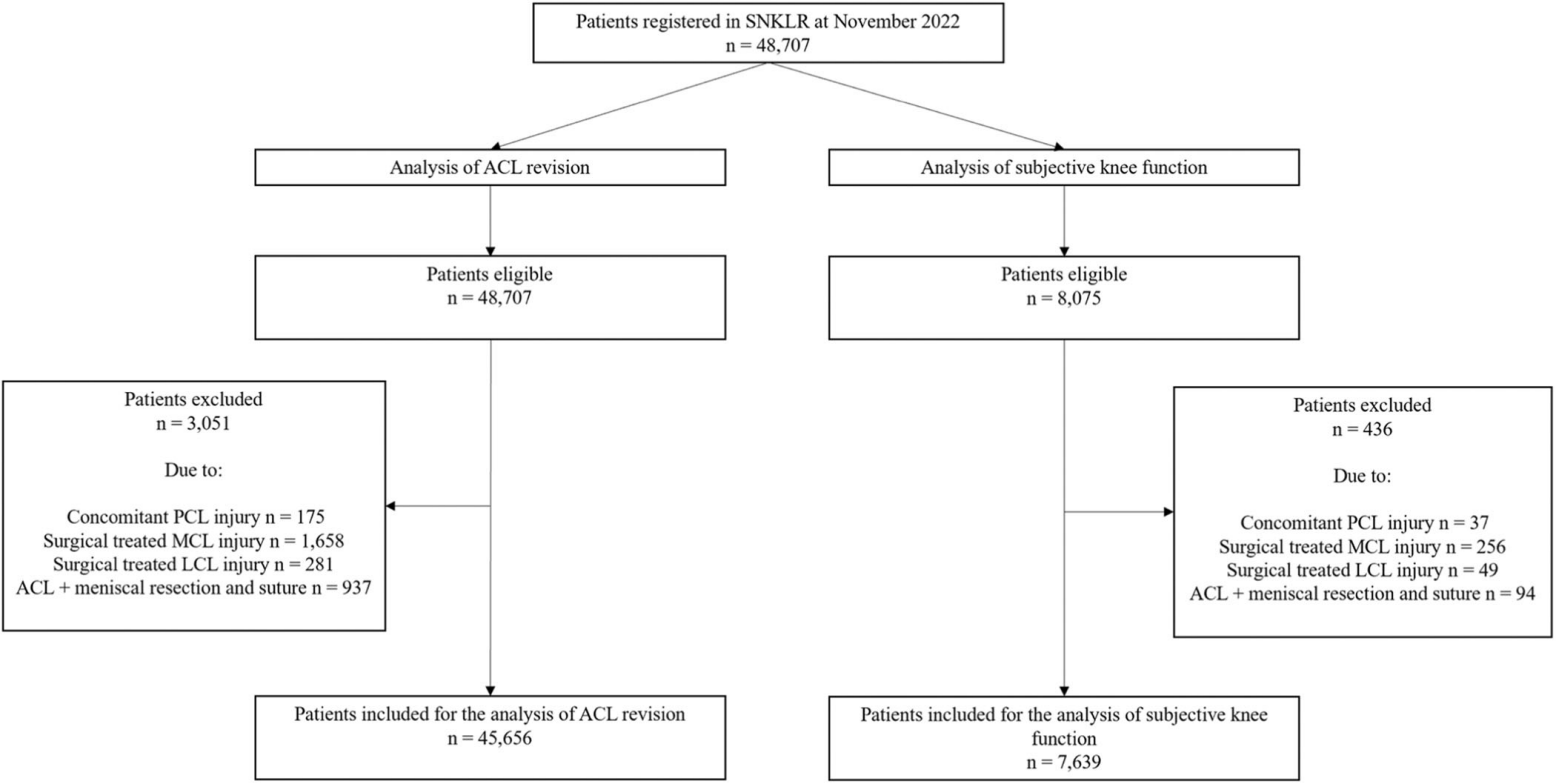
Flowchart of inclusion and exclusion. ACL, anterior cruciate ligament; LCL, lateral collateral ligament; MCL, medial collateral ligament; PCL, posterior cruciate ligament; SNKLR, Swedish National Knee Ligament Registry.

A total of 48,707 patients were registered in SNKLR at November 2022. For the analysis of ACL revision, 45,656 patients met the inclusion criteria, whereas 7639 patients were included in the analysis of subjective knee function following ACL‐R. Figure 2 illustrates the process of inclusion and exclusion.

### Risk for ACL revision

Of the 45,656 patients included in the analysis for ACL revision, the mean age was 27.8 ± 10.2 years and 43.1% of the patients were female. There were 12,038 patients (26.4%) who had a medial meniscal injury, and 10,804 patients (23.7%) who had a lateral meniscal injury. The meniscal injuries were treated with resection in 29.3% of cases and repair in 8.5% of cases. HT autograft was the most common graft used for index ACL‐R (96.0%) compared with PT autograft (4.0%). There were significant differences in age, sex, height, weight, BMI and the presence of medial or lateral meniscal injuries between patients treated with HT autograft and PT autograft. However, the effect sizes were negligible, except for weight which displayed a small effect size (*d* = 0.24, Table 1).

**Table 1:** Demographic data for patients included in the analysis of ACL revision.

	Total (*n* = 45,656)	HT autograft (*n* = 43,817)	PT autograft (*n* = 2469)	Difference between groups, mean (95% CI)	*p* value with effect size (*d)*
Age at index ACL‐R (years)	27.8 (10.2)	27.8 (10.3)	27.0 (9.6)	0.8 (0.5–1.2)	*p* < 0.0001
	(27.7–27.8)	(27.7–27.9)	(26.6–27.3)		*d* = 0.08
Female, *n* (%)	19,678 (43.1)	18,759 (43.4)	919 (37.2)	−6.2 (−8.2 to −4.2)	*p* < 0.0001
					*d* = 0.13
Height (cm)	174.6 (9.3)	174.6 (9.3)	176.0 (9.3)	−1.4 (−2.0 to −0.9)	*p* < 0.0001
	(174.5–174.7)	(174.4–174.7)	(175.4–176.5)		*d* = 0.15
	*n* = 23,508	*n* = 22,399	*n* = 1109		
Weight (kg)	75.9 (14.5)	75.8 (14.4)	79.2 (16.1)	−3.5 (−4.4 to −2.5)	*p* < 0.0001
	(75.7–76.1)	(75.6–75.9)	(78.3–80.2)		*d* = 0.24
	*n* = 23,588	*n* = 22,466	*n* = 1122		
BMI (kg/m^2^)	24.8 (3.7)	24.8 (3.7)	25.5 (4.0)	−0.7 (−0.9 to −0.5)	*p* < 0.0001
	(24.7–24.8)	(24.7–24.8)	(25.2–25.7)		*d* = 0.19
	*n* = 23,482	*n* = 22,373	*n* = 1109		
Medial meniscal injury, *n* (%)	12,038 (26.4)	11,515 (26.7)	523 (21.2)	−5.5 (−7.2 to −3.8)	*p* < 0.0001
					*d* = 0.13
Lateral meniscal injury, *n* (%)	10,804 (23.7)	10,178 (23.6)	626 (25.4)	1.8 (0.0–3.6)	n.s
Meniscal resection, *n* (%)	13,383 (29.3)	12,686 (29.4)	697 (28.2)	−1.1 (−3.0 to 0.7)	n.s
Meniscal repair, *n* (%)	3891 (8.5)	3722 (8.6)	169 (6.8)	−1.8 (−2.8 to −0.7)	*p* = 0.002
					*d* = 0.07

*Note*: Categorical variables are presented with *n* (%). Continuous variables are presented with mean (standard deviation) and a 95% confidence interval.

Abbreviations: ACL, anterior cruciate ligament; ACL‐R, anterior cruciate ligament reconstruction; BMI, body mass index; CI, confidence interval; cm, centimeters; HT, hamstring tendon; kg, kilogram; m, metre; n, numbers; n.s, non‐significant; PT, patellar tendon.

Overall, the rate of ACL revision was 2.4% and 4.9% at 2 and 5 years, respectively. There were no significant differences in the rates of ACL revision between the graft groups regardless of meniscal injury treatment (Table 2).

**Table 2:** The rate of ACL revision between the respective graft groups depending on concomitant meniscal injuries and their treatment 2 and 5 years after ACL‐R.

Treatment	Autograft	No revision	Revision	Difference between groups, mean (95% CI)	*p* value with effect size (d)
Within 2 years		***n*** = **38,836**	***n*** = **918**		
Isolated ACL‐R, *n* (%)		*n* = 21,994	*n* = 536		
	HT	2077 (94.5)	514 (95.9)	−1.4 (−3.2 to 0.4)	n.s
	PT	1217 (5.5)	22 (4.1)		
ACL‐R + meniscal resection, *n* (%)		*n* = 11,415	*n* = 262		
	HT	10,856 (95.1)	246 (93.9)	1.2 (−1.9 to 4.3)	n.s
	PT	559 (4.9)	16 (6.1)		
ACL‐R + meniscal repair, *n* (%)		*n* = 2974	*n* = 57		
	HT	2860 (96.2)	55 (96.5)	−0.3 (−6.0 to 5.4)	n.s
	PT	114 (3.8)	2 (3.5)		
ACL‐R + untreated meniscal injury, *n* (%)		*n* = 2453	*n* = 63		
	HT	2327 (94.9)	61 (96.8)	−2.0 (−7.2 to 3.3)	n.s
	PT	126 (5.1)	2 (3.2)		
Within 5 years		***n*** = **28,810**	***n*** = **1407**		
Isolated ACL‐R, *n* (%)		*n* = 16,586	*n* = 803		
	HT	15,652 (94.4)	767 (95.5)	−1.1 (−2.7 to 0.4)	n.s
	PT	934 (5.6)	36 (4.5)		
ACL‐R + meniscal resection, *n* (%)		*n* = 8675	*n* = 418		
	HT	8258 (95.2)	398 (95.2)	0.0 (−2.2 to 2.2)	n.s
	PT	417 (4.8)	20 (4.8)		
ACL‐R + meniscal repair, *n* (%)		*n* = 1693	*n* = 82		
	HT	1644 (97.1)	78 (95.1)	2.0 (−3.4 to 7.4)	n.s
	PT	49 (2.9)	4 (4.9)		
ACL‐R + untreated meniscal injury, *n* (%)		*n* = 1856	*n* = 104		
	HT	1758 (94.7)	97 (93.3)	1.5 (−4.0 to 6.9)	n.s
	PT	98 (5.3)	7 (6.7)		

Abbreviations: ACL‐R, anterior cruciate ligament reconstruction; CI, confidence interval; HT, hamstring tendon; n, numbers; n.s, non‐significant; PT, patellar tendon.

There was no association of graft choice or meniscal injury treatment on ACL revision up to 5 years after index ACL‐R (Table 3).

**Table 3:** Crude and adjusted hazard ratios of ACL revision up to 5 years after ACL‐R between the study groups.

Groups compared	Crude		Adjusted^a^		Adjusted^a^ (+BMI)	
	Hazard ratio	*p*	Hazard ratio	*p*	Hazard ratio	*p*
Isolated ACL‐R PT autograft vs. isolated ACL‐R HT autograft	0.89 (0.65–1.08)	n.s	0.85 (0.66–1.10)	n.s	0.96 (0.69–1.32)	n.s
ACL‐R + meniscal resection PT autograft vs. ACL‐R + meniscal resection HT autograft	0.85 (0.58–1.24)	n.s	0.84 (0.57–1.22)	n.s	0.53 (0.28–1.00)	n.s
ACL‐R + meniscal repair PT autograft vs. ACL‐R + meniscal repair HT autograft	1.23 (0.58–2.63)	n.s	1.08 (0.50–2.30)	n.s	1.35 (0.49–3.68)	n.s
ACL‐R + untreated meniscal injury PT autograft vs. ACL‐R + untreated meniscal injury HT autograft	1.64 (0.95–2.84)	n.s	1.60 (0.93–2.78)	n.s	1.24 (0.45–3.41)	n.s

*Note*: when adjusting for BMI, approximately half the study population was included as BMI values were missing for the rest.

Abbreviations: ACL‐R, anterior cruciate ligament reconstruction; BMI, body mass index; HT, hamstring tendon; n.s, non‐significant; PT, patellar tendon.

^a^ Adjusted for sex, age, medial meniscal injury and lateral meniscal injury.

### Subjective knee function

Of the 7639 patients included in the analysis of subjective knee function, the mean age was 29.7 ± 11.1 years and 53.7% of patients were female. There were 1983 (26.0%) patients with a medial meniscal injury, and 1598 (20.9%) with a lateral meniscal injury. Meniscal injuries were treated with resection in 29.5% of cases and with repair in 5.4% of cases. HT and PT autografts were used in 94.9% and 5.1% of the primary ACL‐Rs, respectively. There were significant differences in sex, height, weight, BMI, the rate of medial meniscal injuries and meniscal resection between patients treated with HT and PT autografts. The effect sizes for differences in sex, height, weight and BMI were small (*d* = 0.23–0.32). Table 4 displays details in demographics.

**Table 4:** Demographic data of patients included in the analysis of subjective knee function.

	Total (*n* = 7639)	HT autograft (*n* = 7254)	PT autograft (*n* = 385)	Difference between groups, mean (95% CI)	*p* value with effect size (d)
Age at index ACL‐R (years)	29.7 (11.1)	29.8 (11.1)	29.1 (10.6)	0.6 (−0.5 to 1.8)	n.s
	(29.5–30.0)	(29.5–30.0)	(28.1–30.2)		
Female, *n* (%)	4100 (53.7)	3937 (54.3)	163 (42.3)	−11.9 (−17.1 to −6.7)	*p* < 0.0001
					*d* = 0.24
Height (cm)	173.8 (9.1)	173.7 (9.1)	176.0 (8.8)	−2.2 (−3.4 to −1.1)	*p* = 0.0001
	(173.6–174.1)	(173.5–174.0)	(174.9–177.0)		*d* = 0.27
	*n* = 5763	*n* = 5500	*n* = 263		
Weight (kg)	75.1 (14.1)	74.9 (14.0)	79.5 (16.2)	−4.6 (−6.6 to −2.6)	*p* < 0.0001
	(74.7–75.5)	(74.5–75.3)	(77.5–81.4)		*d* = 0.39
	*n* = 5766	*n* = 5502	*n* = 264		
BMI (kg/m^2^)	24.8 (3.6)	24.7 (3.6)	25.6 (4.6)	−0.9 (−1.4 to −0.3)	*p* = 0.004
	(24.7–24.8)	(24.6–24.8)	(25.0–26.1)		*d* = 0.31
	*n* = 5757	*n* = 5494	*n* = 263		
Medial meniscal injury, *n* (%)	1983 (26.0)	1901 (26.2)	82 (21.3)	−4.9 (−9.3 to −0.6)	*p* = 0.004
					*d* = 0.12
Lateral meniscal injury, *n* (%)	1598 (20.9)	1528 (21.1)	70 (18.2)	−2.9 (−7.0 to 1.2)	n.s
Meniscal resection, *n* (%)	2257 (29.5)	2164 (29.8)	93 (24.2)	−5.7 (−10.2 to −1.1)	*p* = 0.02
					*d* = 0.13
Meniscal repair, *n* (%)	409 (5.4)	395 (5.4)	14 (3.6)	−1.8 (−3.9 to 0.3)	n.s

*Note*: Categorical variables presented with *n* (%). Continuous variables presented with mean (standard deviation) and 95% CIs.

Abbreviations: ACL, anterior cruciate ligament; ACL‐R, anterior cruciate ligament reconstruction; BMI, body mass index; CI, confidence interval; cm, centimeters; HT, hamstring tendon; kg, kilogram; m, meter; n, numbers; n.s, non‐significant; PT, patellar tendon.

#### 1 Year after ACL‐R

Patients with isolated ACL‐R scored significantly higher in KOOS‐SR (*p* < 0.0001, d = 0.26) and reached a PASS for KOOS‐SR (48.7% vs. 35.3%, p < 0.0001, *d* = 0.27) to a higher extent when treated with HT autograft compared to PT autograft.

Patients with ACL‐R and meniscal resection scored significantly higher in KOOS‐P, KOOS‐ADL and KOOS‐SR (*∆* = 2.7–12.8, *d* = 0.22–0.49), and reached a PASS to a higher extent in KOOS‐P, KOOS‐ADL and KOOS‐SR (∆ = −11.3% to −22.8%, *d* = 0.25–0.48) when treated with HT autograft compared to PT autograft.

Patients with ACL‐R and meniscal repair scored significantly higher in KOOS‐S (*p* = 0.01, *d* = 0.67) when treated with PT autograft compared to HT autograft. No significant differences in the rate of patients achieving a PASS were observed between the autograft groups in patients with ACL‐R and meniscal repair.

Patients with ACL‐R and untreated meniscal injuries scored significantly higher in KOOS‐S, KOOS‐SR and KOOS‐QoL (*∆* = 8.8–17.6, *d* = 0.50–0.73), and reached a PASS to a higher extent in KOOS‐ADL, KOOS‐SR and KOOS‐QoL (*∆* = −22.7% to −29.5%, *d* = 0.48–0.70) when treated with HT autograft as opposed to PT autograft (Supporting Information S1: Resource 1).

#### 2 Years after ACL‐R

Patients with isolated ACL‐R scored significantly higher in KOOS‐SR (*p* = 0.021, *d* = 0.15) and KOOS‐QoL (*p* = 0.047, *d* = 0.13) when treated with HT autograft compared to PT autograft. There was a greater proportion of patients achieving a PASS for KOOS‐SR in favour of patients treated with HT autograft compared to PT autograft (53.9% vs. 47.2%, *p* = 0.045, *d* = 0.13).

Patients with ACL‐R and meniscal resection scored significantly higher in KOOS‐P (*p* = 0.01, *d* = 0.27) and in KOOS‐SR (*p* = 0.02, *d* = 0.26) when treated with HT autograft compared to PT autograft. In terms of PASS, a greater proportion of patients in the HT autograft group reached the threshold for KOOS‐P, KOOS‐ADL and KOOS‐SR (*∆* = −12.7% to −13.4%, *d* = 0.24–0.29) compared to those treated with PT autograft.

Patients with ACL‐R and meniscal repair scored significantly higher in KOOS‐P, KOOS‐SR and KOOS‐ADL (∆ = −3.9 to −10.3, *d* = 0.32–0.57) when treated with PT autograft compared to HT autograft. However, no significant differences were observed between the autograft groups in the proportion of patients who reached a PASS in the KOOS subscales in patients with ACL‐R and meniscal repair.

Patients with ACL‐R and untreated meniscal injury scored better in KOOS‐S, KOOS‐ADL, KOOS‐SR and KOOS‐QoL (*∆* = 4.8–21.5, *d* = 0.40–0.87) when receiving HT autograft compared to PT autograft. Moreover, patients treated with HT autograft reached a PASS to a higher extent in KOOS‐ADL, KOOS‐SR and KOOS‐QoL (*∆* = −25.7% to −40.3%, *d* = 0.63–0.96) than the PT autograft group (Table 5).

**Table 5:** Subjective knee function assessed with the KOOS subscale scores and the rate of patients achieving a PASS for each subscale 2 years after ACL‐R.

Treatment	KOOS subscale	HT autograft (*n* = 7254)	PT autograft *(n* = 385)	Difference between groups, mean (95% CI)	*p* value with effect size (d)
**Isolated ACL‐R**		***n*** = **4211**	***n*** = **252**		
	KOOS‐P	86.0 (15.1)	85.7 (15.4)	0.3 (−1.6 to 2.3)	n.s
		(85.6–86.5)	(83.8–87.6)		
	KOOS‐S	80.3 (17.3)	79.2 (17.7)	1.1 (−1.1 to 3.3)	n.s
		(79.7–80.8)	(77.0–81.3)		
	KOOS‐ADL	92.4 (12.4)	91.8 (12.7)	0.6 (−1.0 to 2.2)	n.s
		(92.0–92.8)	(90.2–93.4)		
	KOOS‐SR	68.5 (26.4)	64.5 (26.5)	4.0 (0.6–7.3)	*p* = 0.02
		(67.7–69.3)	(61.2–67.8)		*d* = 0.15
	KOOS‐QoL	64.3 (23.3)	61.3 (23.2)	3.0 (0.04–6.0)	*p* = 0.047
		(63.6–65.0)	(58.4–64.1)		*d* = 0.13
	**PASS, *n* (%)**				
	KOOS‐P	2170 (51.5)	134 (53.2)	1.6 (−4.9 to 8.2)	n.s
	KOOS‐S	3789 (90.0)	223 (88.5)	−1.5 (−5.7 to 2.8)	n.s
	KOOS‐ADL	1550 (36.8)	83 (32.9)	−3.9 (−10.1 to 2.3)	n.s
	KOOS‐SR	2271 (53.9)	119 (47.2)	−6.7 (−13.3 to −0.2)	*p* = 0.045
					*d* = 0.13
	KOOS‐QoL	2262 (53.7)	120 (47.6)	−6.1 (−12.7 to 0.5)	n.s
**ACL‐R + meniscal resection**		***n*** = **2164**	***n*** = **93**		
	KOOS‐P	86.5 (15.0)	82.5 (16.0)	4.1 (1.0–7.2)	*p* = 0.01
		(85.9–87.2)	(79.2–85.8)		*d* = 0.27
	KOOS‐S	80.1 (17.2)	77.5 (17.2)	2.6 (−1.0 to 6.2)	n.s
		(79.4–80.8)	(74.0–81.0)		
	KOOS‐ADL	92.9 (12.5)	90.5 (12.5)	2.0 (−0.6 to 4.6)	n.s
		(92.0–93.0)	(87.9–93.1)		
	KOOS‐SR	68.2 (26.6)	61.3 (26.7)	6.9 (1.3–12.4)	*p* = 0.02
		(67.1–69.3)	(55.8–66.9)		*d* = 0.26
	KOOS‐QoL	64.0 (22.6)	59.3 (23.4)	4.6 (−0.1 to 9.3)	n.s
		(63.0–64.9)	(54.5–64.2)		
	**PASS, *n* (%)**				
	KOOS‐P	1148 (53.0)	37 (39.8)	−13.3 (−24.0 to −2.5)	*p* = 0.02
					*d* = 0.27
	KOOS‐S	1963 (90.7)	84 (90.3)	−0.4 (−7.1 to 6.3)	n.s
	KOOS‐ADL	801 (37.0)	22 (23.7)	−13.4 (−22.8 to −3.9)	*p* = 0.01
					*d* = 0.29
	KOOS‐SR	1146 (53.0)	38 (40.9)	−12.7 (−22.9 to −1.3)	*p* = 0.03
					*d* = 0.24
	KOOS‐QoL	1132 (52.3)	43 (46.2)	−6.1 (−17.0 to 4.8)	n.s
**ACL‐R + meniscal repair**		***n*** = **395**	***n*** = **14**		
	KOOS‐P	84.8 (15.3)	89.9 (8.2)	−5.1 (−10.0 to −0.17)	*p* = 0.04
		(83.3–86.3)	(85.1–94.6)		*d* = 0.34
	KOOS‐S	76.2 (18.2)	86.5 (16.0)	−10.3 (−20.0 to −0.6)	*p* = 0.04
		(74.4–78.0)	(77.2–95.7)		*d *= 0.57
	KOOS‐ADL	92.6 (12.4)	96.4 (4.7)	−3.9 (−6.8 to −0.9)	*p* = 0.02
		(91.3–93.8)	(93.7–99.1)		*d* = 0.32
	KOOS‐SR	65.9 (26.8)	68.6 (23.1)	−2.7 (−16.9 to 11.6)	n.s
		(63.6–68.6)	(55.2–81.9)		
	KOOS‐QoL	60.7 (23.2)	59.8 (25.9)	0.9 (−11.5 to 13.4)	n.s
		(58.4–63.0)	(44.9–74.8)		
	**PASS, *n* (%)**				
	KOOS‐P	187 (47.3)	7 (50.0)	2.7 (−27.7 to 33.0)	n.s
	KOOS‐S	339 (85.8)	13 (92.9)	7.1 (−10.6 to 24.7)	n.s
	KOOS‐ADL	143 (36.2)	6 (42.9)	6.7 (−23.4 to 36.7)	n.s
	KOOS‐SR	196 (49.6)	7 (50.0)	0.4 (−30.0 to 30.7)	n.s
	KOOS‐QoL	181 (45.8)	7 (50.0)	4.2 (−26.2 to 34.5)	n.s
**ACL‐R + untreated meniscal injury**		***n*** = **484**	***n*** = **26**		
	KOOS‐P	86.6 (13.9)	81.7 (15.2)	4.9 (−0.6 to 10.4)	n.s
		(85.4–87.9)	(75.6–87.9)		
	KOOS‐S	80.4 (17.0)	70.3 (17.1)	10.1 (3.3–16.8)	*p* = 0.004
		(78.9–81.9)	(63.4–77.2)		*d* = 0.59
	KOOS‐ADL	93.0 (12.0)	88.2 (10.6)	4.8 (0.1–9.5)	*p* = 0.047
		(91.9–94.1)	(84.0–92.5)		*d* = 0.40
	KOOS‐SR	69.4 (24.8)	47.9 (25.3)	21.5 (11.7–31.3)	*p* < 0.0001
		(67.1–71.6)	(37.7–58.1)		*d* = 0.87
	KOOS‐QoL	63.8 (21.9)	46.9 (18.7)	17.0 (8.4–25.5)	*p* = 0.0001
		(61.9–65.8)	(39.3–54.4)		*d* = 0.78
	**PASS, *n* (%)**				
	KOOS‐P	252 (52.1)	9 (34.6)	−17.5 (−38.3 to 3.4)	n.s
	KOOS‐S	435 (89.9)	20 (76.9)	−13.0 (−31.4 to 5.5)	n.s
	KOOS‐ADL	180 (37.2)	3 (11.5)	−25.7 (−40.7 to −10.6)	*p* = 0.009
					*d* = 0.63
	KOOS‐SR	261 (53.9)	4 (15.4)	−38.5 (−55.1 to −22.0)	*p* = 0.0002
					*d* = 0.89
	KOOS‐QoL	251 (51.9)	3 (11.5)	−40.3 (−55.4 to −25.2)	*p* < 0.0001
					*d* = 0.96

*Note*: Categorical variables presented with *n* (%). Continuous variables presented with mean (standard deviation) and 95% CIs.

Abbreviations: ACL‐R, anterior cruciate ligament reconstruction; CI, confidence interval; HT, hamstring tendon; KOOS, Knee Injury and Osteoarthritis Outcome Score; KOOS‐ADL, Subscale activities in daily living; KOOS‐P, subscale pain; KOOS‐S, subscale symptoms; KOOS‐SR, subscale sports and recreational; KOOS‐QoL, subscale quality of life; n, numbers; n.s, non‐significant; PASS, patient acceptable symptom state; PT, patellar tendon.

#### 5 Years after ACL‐R

Patients with isolated ACL‐R scored significantly higher in KOOS‐SR (*p* = 0.03, *d* = 0.14) and reached a PASS to a greater extent in KOOS‐SR (56.6% vs. 47.2%, *p* = 0.004, *d* = 0.19) when treated with HT autograft compared to PT autograft.

Patients with ACL‐R and meniscal resection scored significantly higher in KOOS‐SR (*p* = 0.002, *d* = 0.33) and KOOS‐QoL (*p* = 0.008, *d* = 0.28) when treated with HT autograft compared to PT autograft. Moreover, patients with HT autograft reached a PASS to a greater extent in KOOS‐P, KOOS‐ADL, KOOS‐SR and KOOS‐QoL (*∆* = −12.0% to −15.8%, *d* = 0.24–0.32) compared to patients treated with PT autograft.

Patients with ACL‐R and meniscal repair scored significantly higher in KOOS‐ADL (*p* = 0.03, *d* = 0.36) and reached a pass to a greater extent in the KOOS‐ADL (71.4% vs. 39.2%, *p* = 0.03, *d* = 0.68) when treated with PT autograft compared to HT autograft.

Patients with ACL‐R and untreated meniscal injuries reported better scores for all KOOS subscales (*∆* = 8.3–18.6, *d* = 0.67–0.77) and reached a PASS (*∆* = −22.3% to −30.6%, *d* = 0.54–0.72) to a greater extent in all KOOS subscales when treated with HT autograft compared to PT autograft (Table 6).

**Table 6:** Subjective knee function assessed with the KOOS subscale scores and the rate of patients achieving a PASS for each subscale 5 years after ACL‐R.

Treatment	KOOS subscale	HT autograft (n = 7254)	PT autograft (n = 385)	Difference between groups, mean (95% CI)	*p* value with effect size (d)
**Isolated ACL‐R**		***n*** = **4211**	***n*** = **252**		
	KOOS‐P	87.1 (15.4)	86.7 (15.7)	0.4 (−1.6 to 2.3)	n.s
		(86.6–87.5)	(84.8–88.7)		
	KOOS‐S	81.9 (17.4)	81.9 (17.3)	−0.01 (−2.2 to 2.2)	n.s
		(81.4–82.4)	(79.8–84.1)		
	KOOS‐ADL	92.8 (12.6)	92.0 (13.9)	−0.7 (−1.0 to 2.5)	n.s
		(92.4–93.1)	(90.3–93.7)		
	KOOS‐SR	70.0 (27.4)	66.1 (26.8)	3.9 (0.5–7.4)	*p* = 0.03
		(66.3–67.8)	(62.8–69.4)		*d* = 0.14
	KOOS‐QoL	67.0 (24.3)	64.9 (24.3)	2.2 (−0.9 to 5.3)	n.s
		(66.3–67.8)	(61.8–67.9)		
	**PASS, *n* (%)**				
	KOOS‐P	2378 (56.5)	143 (56.7)	0.3 (−6.2 to 6.8)	n.s
	KOOS‐S	3818 (90.7)	226 (89.7)	−1.0 (−5.1 to 3.1)	n.s
	KOOS‐ADL	1739 (41.3)	93 (36.9)	−4.4 (−10.7 to 2.0)	n.s
	KOOS‐SR	2385 (56.6)	119 (47.2)	−9.4 (−16.0 to −2.9)	*p* = 0.004
					*d* = 0.19
	KOOS‐QoL	2487 (59.1)	139 (55.2)	−3.9 (−10.4 to 2.6)	n.s
**ACL‐R + meniscal resection**		***n*** = **2164**	***n*** = **93**		
	KOOS‐P	87.2 (15.6)	85.1 (14.2)	2.1 (−1.1 to 5.3)	n.s
		(86.8–87.9)	(82.2–88.0)		
	KOOS‐S	81.7 (17.3)	80.0 (15.8)	1.7 (−1.8 to 5.3)	n.s
		(81.0–82.4)	(76.7–83.2)		
	KOOS‐ADL	92.7 (12.9)	91.1 (12.0)	1.5 (−1.1 to 4.2)	n.s
		(92.1–93.2)	(88.7–93.6)		
	KOOS‐SR	69.9 (27.1)	60.9 (26.8)	9.1 (3.4–14.7)	*p* = 0.002
		(68.8–71.1)	(55.3–66.4)		*d* = 0.33
	KOOS‐QoL	67.0 (23.9)	60.2 (24.4)	6.8 (1.8–11.7)	*p* = 0.008
		(66.0–68.0)	(55.2–65.2)		*d* = 0.28
	**PASS, *n* (%)**				
	KOOS‐P	1236 (57.1)	40 (43.0)	−14.1 (−24.9 to −3.3)	*p* = 0.01
					*d* = 0.28
	KOOS‐S	1974 (91.2)	87 (93.5)	2.3 (−3.4 to 8.0)	n.s
	KOOS‐ADL	903 (41.7)	26 (28.0)	−13.8 (−23.7 to −3.9)	*p* = 0.01
					*d* = 0.29
	KOOS‐SR	1203 (55.6)	37 (39.8)	−15.8 (−26.5 to −5.1)	*p* = 0.004
					*d* = 0.32
	KOOS‐QoL	1260 (58.2)	43 (46.2)	−12.0 (−22.9 to −1.1)	*p* = 0.03
					*d* = 0.24
**ACL‐R + meniscal repair**		***n*** = **395**	***n*** = **14**		
	KOOS‐P	85.3 (15.4)	92.3 (10.6)	−7.0 (−15.1 to 1.2)	n.s
		(83.8–86.8)	(86.2–98.4)		
	KOOS‐S	79.1 (17.3)	86.7 (12.6)	−7.6 (−16.8 to 1.6)	n.s
		(77.4–80.8)	(79.5–94.0)		
	KOOS‐ADL	92.9 (11.8)	97.1 (6.0)	−4.1 (−7.8 to −0.5)	*p* = 0.03
		(91.8–94.1)	(93.6–100.5)		*d* = 0.36
	KOOS‐SR	66.4 (27.9)	71.1 (22.8)	−4.6 (−19.5 to 10.2)	n.s
		(63.7–69.2)	(57.9–84.2)		
	KOOS‐QoL	62.5 (23.8)	65.2 (27.6)	−2.7 (−15.5 to 10.0)	n.s
		(60.1–64.8)	(49.2–81.1)		
	**PASS, *n* (%)**				
	KOOS‐P	192 (48.6)	9 (64.3)	15.7 (−13.6 to 45.0)	n.s
	KOOS‐S	349 (88.4)	13 (92.9)	4.5 (−13.1 to 22.1)	n.s
	KOOS‐ADL	155 (39.2)	10 (71.4)	32.3 (4.3–60.0)	*p* = 0.03
					*d* = 0.68
	KOOS‐SR	200 (50.6)	8 (57.1)	6.5 (−23.6 to 36.6)	n.s
	KOOS‐QoL	197 (49.9)	7 (50.0)	0.1 (−30.2 to 30.5)	n.s
**ACL‐R + untreated meniscal injury**		***n*** = **484**	***n*** = **26**		
	KOOS‐P	87.3 (14.2)	77.1 (16.3)	10.2 (4.5–15.9)	*p* = 0.0005
		(86.1–88.6)	(70.6–83.7)		*d* = 0.71
	KOOS‐S	81.7 (16.7)	68.8 (18.9)	12.9 (6.2–19.6)	*p* = 0.0002
		(80.2–83.2)	(61.2–76.5)		*d* = 0.77
	KOOS‐ADL	92.9 (11.5)	84.7 (15.7)	8.3 (1.9–14.7)	*p* = 0.01
		(91.9–94.0)	(78.3–91.0)		*d* = 0.70
	KOOS‐SR	69.5 (26.2)	51.0 (24.0)	18.6 (8.3–28.9)	*p* = 0.0004
		(67.2–71.9)	(41.3–60.7)		*d* = 0.71
	KOOS‐QoL	65.1 (24.0)	50.2 (21.2)	15.8 (6.4–25.3)	*p* = 0.001
		(63.9–68.2)	(41.7–58.8)		*d* = 0.67
	**PASS, *n* (%)**				
	KOOS‐P	275 (56.8)	8 (30.8)	−26.0 (−46.4 to −5.7)	*p* = 0.02
					*d* = 0.54
	KOOS‐S	443 (91.5)	18 (69.2)	−22.3 (−42.2 to −2.4)	*p* = 0.003
					*d* = 0.59
	KOOS‐ADL	201 (41.5)	3 (11.5)	−30.0 (−45.1 to −14.9)	*p* = 0.003
					*d* = 0.72
	KOOS‐SR	260 (53.7)	6 (23.1)	−30.6 (−49.5 to −11.8)	*p* = 0.004
					*d* = 0.66
	KOOS‐QoL	275 (56.8)	8 (30.8)	−26.0 (−46.4 to −5.7)	*p* = 0.02
					*d* = 0.54

*Note*: Categorical variables presented with *n* (%). Continuous variables presented with mean (standard deviation) and 95% CIs.

Abbreviations: ACL‐R, anterior cruciate ligament reconstruction; CI, confidence interval; HT, hamstring tendon; KOOS, Knee Injury and Osteoarthritis Outcome Score; KOOS‐ADL, subscale activities in daily living; KOOS‐P, subscale pain; KOOS‐QoL, subscale quality of life; KOOS‐S, subscale symptoms; KOOS‐SR, subscale sports and recreational; n, numbers; n.s, non‐significant; PASS, patient acceptable symptom state; PT, patellar tendon.

## DISCUSSION

The most important finding of the present study was that the rate of ACL revision was not significantly different between patients who underwent ACL‐R with HT or PT autograft and had a concurrent meniscal injury, irrespective of meniscal injury treatment. In addition, a greater proportion of patients with HT autograft perceived a superior postoperative knee function as they achieved a PASS in KOOS subscales to a greater extent compared with patients with PT autograft when the meniscus was resected, or when the meniscus injury was left untreated.

### Rate of ACL revision

No significant difference in ACL revision rates was evident between graft selection, irrespective of concurrent meniscal injuries and their treatment within 5 years after ACL‐R in our study encompassing 45,656 patients. Previous studies have reported a greater risk for ACL revision for patients treated with HT autografts compared to PT autografts [31, 37, 41, 47]. This increased risk may partly be due to an increased knee laxity for HT autografts compared to PT autografts [10, 38]. The knee laxity may be greater in cases of ACL‐R with additional medial meniscal resection compared to isolated ACL‐R or ACL‐R with medial meniscal repair [16]. The menisci play a crucial role in controlling knee laxity, with the medial meniscus limiting excessive anterior tibial translation and the lateral meniscus controlling rotational laxity [18, 29]. Thus, the menisci are vital for reducing excessive stress on the ACL by mitigating excessive anterior and rotational motions [18]. Speculatively, using HT autograft for ACL‐R alongside meniscal resection may magnify the rate of ACL revision compared with PT autografts. Despite this logical premise, the present study found that graft selection did not impact the rate of ACL revision in patients with concurrent meniscal injuries irrespective of its treatment within 5 years after ACL‐R.

### Subjective knee function

The goal following ACL‐R is to attain a satisfactory knee function. A meta‐analysis comparing outcomes between HT and PT autografts reported no significant differences in PROMs [44]. Another systematic review reported that HT autograft and the absence of concurrent injuries, such as meniscal injuries, were associated with superior PROMs after ACL‐R [20]. In the present study, patients who underwent ACL‐R treated with HT autograft and concurrent meniscal resection achieved a significantly greater rate of PASS in the KOOS‐P subscale than the corresponding group with PT autograft. This was observed at the 1 (48.9% vs. 32.3%), 2 (53.0% vs. 39.8%) and 5‐year follow‐up (57.1% vs. 43.0%). Previous studies have reported a greater prevalence of anterior knee pain associated with PT autografts [27, 50], which may have potentially contributed to the inferior PASS rate for KOOS‐P among patients treated with PT autografts compared with patients with HT autografts. However, differences in achieving PASS in terms of the KOOS‐P were mainly noticeable between graft selection among patients treated with meniscal resection. Furthermore, patients with ACL‐R using an HT autograft and concurrent meniscal resection achieved a PASS in the Sport/Rec subscale to a greater extent than patients with PT autografts and meniscal resection at 1 (50.8% vs. 28.0%), 2 (53.0% vs. 40.9%) and 5 years (55.6% vs 39.8%). Interestingly, the significantly lower rates of achieving PASS in KOOS‐SR were not observed in patients with ACL‐R treated with PT autograft and meniscal repair compared with patients treated with HT autograft and meniscal repair. Meniscal resection has previously been associated with inferior PROMs, reduced activity levels and increased rate of postoperative knee osteoarthritis compared to meniscal repair [30, 32, 45]. The use of the PT autograft has also been related to knee extensor strength deficit compared with HT autografts [43], and being symmetrical in knee extensor strength has been associated with the achievement of PASS in KOOS subscales [13] and may reduce the odds of symptomatic knee osteoarthritis [4]. Data suggest that initial signs of knee osteoarthritis, such as bone marrow, cartilage and meniscal lesions, and osteophytes, may manifest as early as one year after ACL‐R in individuals who return to sport prematurely with compromised lower limb function [11]. However, whether our cohort exhibited early signs of knee osteoarthritis at 1 and 2 years, impacting their pain and activity levels, is unknown. The reasons behind the lower proportion of patients treated with PT autograft and meniscal resection or untreated meniscal injury achieving PASS in KOOS‐P and KOOS‐SR—whether due to anterior knee pain, diminished knee extensor strength, early onset of knee osteoarthritis or other causes—remain elusive and requires further investigation.

### Limitations

The current study presents several limitations. One notable concern is potentially missing ACL revision registrations, leading to an underestimation of the true rate of ACL revisions. Nevertheless, the observed rates of ACL revision in our study align closely with the general revision rates reported across national ACL registry studies [33]. Another limitation is the lack of activity level data in the SNKLR, an important factor known to influence the rate of second ACL injuries [49]. This might have biased our analysis of ACL revision, for example, patients may not have been active preinjury or returned to knee‐strenuous activities postoperatively. In addition, patients who undergo ACL‐R within 3 months of injury might have an increased risk of ACL revision [40]. The timing of ACL‐R was not accounted for in our analysis of ACL revision.

Moreover, our analysis of graft selection for different patient subgroups based on the presence of a meniscal injury and its treatment yielded relatively small patient cohorts in the PT autograft treatment group. This raises (1) the risk of Type II errors—false negatives due to inadequate sample size, and (2) potential bias in the estimation of differences due to lower precision in the estimated mean and standard deviation for the smaller groups. Furthermore, our study did not consider the specific localisation of the meniscal injuries. Variations such as smaller vertical longitudinal ruptures (<10 mm) vs. radial ruptures might have different treatments and outcomes [39]. Additionally, we did not identify and exclude patients with meniscal repair failure, which may lead to inferior KOOS [9]. Neither did we incorporate assessments of knee extensor strength between the graft groups when evaluating both the rate of ACL revision and the achievement of PASS. This could have provided valuable insight into our analysis. The cut‐offs for achieving PASS in the KOOS subscales applied in the present study were taken from the study by Muller et al. [28], hence if these cut‐offs are accurate for the patients in the present study is unknown since the patients included were not asked if they perceived their knee function as acceptable. The KOOS is limited in the content validity and structural validity for patients after ACL‐R [17], which requires further caution in the interpretation of our results. Finally, the reasons underlying the choice of graft remain unknown in our study. This lack of information may introduce indication bias, as the decision‐making process behind graft selection could have influenced the outcomes observed.

## CONCLUSION

Graft selection was not associated with ACL revision in patients with ACL‐R and concurrent meniscal injury, regardless of meniscal injury treatment. Superior subjective knee function was reported by patients who underwent ACL‐R with HT autograft compared with PT autograft, where the injured meniscus was resected or left in situ.

## AUTHOR CONTRIBUTIONS

Johan Högberg and Lina Petersson drafted the initial version of the manuscript. Johan Högberg, Lina Petersson and Eric Hamrin Senorski contributed substantially to the acquisition of the data and analysis of the data, and they are responsible for drafting the manuscript and revising it critically for important intellectual content. Bálint Zsidai, Alexandra Horvath, Riccardo Cristiani and Kristian Samuelsson made large contributions to the revision and design of the work. Johan Högberg and Eric Hamrin Senorski are responsible for the concept of design.

## CONFLICT OF INTEREST STATEMENT

Kristian Samuelsson is a board member of Getinge AB. The remaining authors declare no conflicts of interest.

## ETHICS STATEMENT

All patients received written and oral information to participate in the Swedish National Knee Ligament Registry. The study was approved by the Swedish Ethical Review Authority (2022‐00913‐01).

## Supporting information

Supporting information.

## Data Availability

Data are available on reasonable request.
