# Supplementary material for: No difference in ACL revision rates between hamstring and patellar tendon autograft in patients with ACL‐R and a concurrent meniscal injury irrespective of meniscal treatment
Source: Knee Surg Sports Traumatol Arthrosc. 2025 Jan 23;33(7):2422–37. doi: 10.1002/ksa.12592 (PMC12205421; doi:10.1002/ksa.12592)
Supplement: Supplementary file 1 — Supporting information. [file KSA-33-2422-s001.docx]

**Online Resource 1**

*Online Resource 1.*  *Subjective knee function assessed with the* *KOOS subscale scores and the rate of patients achieving PASS for each subscale one year after ACL-R.*

| **Treatment** | **KOOS subscale** | **HT**  **autograft**  **(n = 7,254)** | **PT**  **autograft**  **(n = 385)** | **Difference between groups Mean (95% CI)** | | **p-value with effect** s**ize (d)** |
| --- | --- | --- | --- | --- | --- | --- |
| **Isolated ACL-R** |  | **n = 4,211** | **n = 252** |  | |  |
|  | *KOOS-P* | 85.6 (14.5) (85.2; 86.1) | 83.6 (16.4) (81.5; 85.6) | 2.1 (-0.0; 4.2) | | n.s |
|  | *KOOS-S* | 78.6 (17.1) (78.1; 79.1) | 78.5 (18.4) (76.2; 80.8) | 0.2 (-2.0; 2.3) | | n.s |
|  | *KOOS-ADL* | 92.5 (11.9) (92.2; 92.9) | 91.4 (12.2) (89.8; 92.9) | 1.2 (-0.4; 2.7) | | n.s |
|  | *KOOS-SR* | 66.3 (25.9) (65.5; 67.1) | 59.5 (25.6) (56.3; 62.6) | 6.8 (3.5; 10.1) | | p <.001 d = 0.26 |
|  | *KOOS-QoL* | 61.0 (22.4) (60.3; 61.6) | 58.3 (21.8) (56.3; 62.6) | 2.6 (-0.2; 5.5) | | n.s |
|  | **PASS, n (%)** |  |  |  | |  |
|  | *KOOS-P* | 2,042 (48.5) | 111 (44.0) | -4.4 (-11.0; 2.1) | | n.s |
|  | *KOOS-S* | 3,768 (89.5) | 218 (86.5) | -3.0 (-7.5; 1.6) | | n.s |
|  | *KOOS-ADL* | 1,403 (33.3) | 80 (31.7) | -1.6 (-7.7; 4.6) | | n.s |
|  | *KOOS-SR* | 2,051 (48.7) | 89 (35.3) | -13.4 (-19.7; -7.1) | | p <.0001 d = 0.27 |
|  | *KOOS-QoL* | 1,948 (46.3) | 110 (43.7) | -2.6 (-9.1; 3.9) | | n.s |
| **ACL-R + Meniscal resection** |  | **n = 2,164** | **n = 93** |  | |  |
|  | *KOOS-P* | 86.1 (14.4) (85.5; 86.7) | 81.1 (14.4) (78.2; 84.1) | 5.0 (2.0; 8.0) | | p = 0.001 d = 0.35 |
|  | *KOOS-S* | 78.4 (16.9) (77.7; 79.1) | 75.2 (17.2) (71.6; 78.7) | 3.2 (-0.3; 6.8) | | n.s |
|  | *KOOS-ADL* | 92.5 (11.8) (92.0; 93.0) | 89.8 (12.2) (87.3; 92.3) | 2.7 (0.2; 5.1) | | p = 0.04 d = 0.22 |
|  | *KOOS-SR* | 66.8 (26.0) (65.7; 67.9) | 54.0 (25.27) (48.7; 59.3) | 12.8 (7.4; 18.2) | | p < .0001 d = 0.49 |
|  | *KOOS-QoL* | 61.1 (21.9) (60.1; 62.0) | 57.2 (20.7) (52.9; 61.4) | 3.9 (-0.7; 8.4) | | n.s |
|  | **PASS, n (%)** |  |  |  | |  |
|  | *KOOS-P* | 1,059 (48.9) | 30 (32.3) | -16.7 (-27.0; -6.4) | | p = 0.002 d = 0.34 |
|  | *KOOS-S* | 1,927 (89.0) | 82 (88.2) | -0.9 (-8.1; 6.4) | | n.s |
|  | *KOOS-ADL* | 733 (33.9) | 21 (22.6) | -11.3 (-20.6; -2.0) | | p = 0.03 d = 0.25 |
|  | *KOOS-SR* | 1,099 (50.8) | 26 (28.0) | -22.8 (-32.8; -12.9) | | p < .0001 d = 0.48 |
|  | *KOOS-QoL* | 985 (45.5) | 36 (38.7) | -6.8 (-17.5; 3.9) | | n.s |
| **ACL-R + Meniscal repair** |  | **n = 395** | **n = 14** |  | |  |
|  | *KOOS-P* | 84.8 (14.4) (83.4; 86.2) | 89.5 (8.8) (84.4; 94.6) | -4.7 (-9.9; 0.6) | | n.s |
|  | *KOOS-S* | 75.3 (17.0) (73.7; 77.0) | 86.7 (11.2) (80.3; 93.2) | -11.4 (-20.4; -2.4) | | p = 0.01 d = 0.67 |
|  | *KOOS-ADL* | 92.7 (11.2) (91.5; 93.8) | 95.4 (5.7) (92.1; 98.7) | -2.7 (-6.2; 0.7) | | n.s |
|  | *KOOS-SR* | 62.9 (26.1) (60.4; 65.5) | 66.8 (27.9) (50.7; 82.9) | -3.5 (-17.8; 10.2) | | n.s |
|  | *KOOS-QoL* | 58.2 (22.2) (56.0; 60.4) | 61.2 (20.5) (49.3; 73.0) | -3.0 (-14.8; 8.9) | | n.s |
|  | **PASS, n (%)** |  |  |  | |  |
|  | *KOOS-P* | 173 (43.8) | 8 (57.1) | 13.3 (-16.7; 43.4) | | n.s |
|  | *KOOS-S* | 337 (85.3) | 14 (100.0) | 14.7 (7.5; 21.9) | | n.s |
|  | *KOOS-ADL* | 120 (30.4) | 5 (35.7) | 5.3 (-23.9; 34.5) | | n.s |
|  | *KOOS-SR* | 175 (44.3) | 8 (57.1) | 12.8 (-17.2; 42.9) | | n.s |
|  | *KOOS-QoL* | 163 (41.3) | 6 (42.9) | 1.6 (-28.5; 31.7) | | n.s |
| **ACL-R + untreated meniscal injury** |  | **n = 484** | **n = 26** |  | |  |
|  | *KOOS-P* | 86.2 (14.4) (85.60; 87.5) | 82.4 (14.1) (76.7; 88.1) | 3.9 (-1.8; 9.6) | | n.s |
|  | *KOOS-S* | 78.6 (17.4) (77.0; 80.2) | 69.8 (20.5) (61.5; 78.1) | 8.8 (1.9; 15.8) | | p = 0.01 d = 0.50 |
|  | *KOOS-ADL* | 92.8 (12.0) (91.7; 93.9) | 88.9 (11.9) (84.1; 93.7) | 3.9 (-0.8; 8.6) | | n.s |
|  | *KOOS-SR* | 67.6 (24.8) (65.4; 69.8) | 50.0 (26.2) (39.4; 60.6) | 17.6 (7.7; 27.4) | p = 0.0005 d = 0.71 | |
|  | *KOOS-QoL* | 61.5 (21.0) (59.7; 63.4) | 46.2 (22.2) (37.2; 55.1) | 15.4 (7.1; 23.7) | p = 0.0003 d = 0.73 | |
|  | **PASS, n (%)** |  |  |  | |  |
|  | *KOOS-P* | 242 (50.0) | 9 (34.6) | -15.4 (-36.2; 5.5) | | n.s |
|  | *KOOS-S* | 434 (89.7) | 21 (80.8) | -8.9 (-26.3; 8.5) | | n.s |
|  | *KOOS-ADL* | 169 (34.9) | 2 (7.7) | -27.2 (-26.3; -8.5) | | p = 0.004 d = 0.70 |
|  | *KOOS-SR* | 240 (49.6) | 7 (26.9) | -22.7 (-42.3; -3.0) | | p = 0.04 d = 0.48 |
|  | *KOOS-QoL* | 217 (44.8) | 4 (15.4) | -29.5 (-46.0; -12.9) | | p = 0.004 d = 0.68 |

*Categorical variables presented with n (%). Continuous variables presented with mean (standard deviation) and 95% confidence intervals. ACL-R = Anterior Cruciate Ligament Reconstruction; CI = Confidence interval; HT = Hamstring tendon; KOOS = Knee injury and osteoarthritis outcome score; KOOS-P = Subscale pain; KOOS-S = Subscale symptoms; KOOS-ADL = Subscale activities in daily living; KOOS-SR = Subscale sports and recreational; KOOS-QoL = Subscale quality of life; n = numbers; n.s = non-significant; PASS = Patient acceptable symptom state; PT = Patellar tendon.*
